# Translation and validation of caring behaviors inventory among nurses in Iran

**DOI:** 10.1371/journal.pone.0254317

**Published:** 2021-07-09

**Authors:** Raziyeh Ghafouri, Malihe Nasiri, Foroozan Atashzadeh-Shoorideh, Faraz Tayyar-Iravanlou, Zahra Rahmaty

**Affiliations:** 1 Medical and Surgical Department, School of Nursing & Midwifery, Shahid Beheshti University of Medical Science, Tehran, Iran; 2 Department of Basic Sciences, School of Nursing & Midwifery, Shahid Beheshti University of Medical Science, Tehran, Iran; 3 Department of Psychiatric Nursing and Management, School of Nursing & Midwifery, Shahid Beheshti University of Medical Sciences, Tehran, Iran; 4 Student Research Committee, School of Nursing and Midwifery, Shahid Beheshti University of Medical Sciences, Tehran, Iran; 5 Center for Health Outcome Research, School of Nursing, University of Maryland, Baltimore, Maryland, United States of America; Tabriz University of Medical Sciences, ISLAMIC REPUBLIC OF IRAN

## Abstract

**Background and objectives:**

Nurses’ caring behaviors, professional activities, and behaviors for the benefit of patients, influence patients’ perception of care and satisfaction with the quality of care provided. Caring behaviors of nurses are contextual and various factors such as patients’ social structure, lifestyle, culture, and interests, as well as their biographical, social, and physiological characteristics, can influence perceptions of caring behaviors of nurses, as caring behaviors are an interactive and mental process between patients and nurses. This study was conducted to provide a transcultural translation and psychometric analysis of Caring Behaviors Inventory (CBI) among nurses in Iran.

**Methodology:**

Transcultural translation of the 16-item CBI was performed. Then, face validity (qualitative), content validity (quantitative and qualitative), and construct validity were examined in a cross-sectional study of 509 patients. A demographic questionnaire and the 16-item CBI were sent to enrolled patients via online questionnaires. The reliability of the instrument was assessed by internal consistency using Cronbach’s alpha. Then, construct validity of the single factor CBI was assessed using Confirmatory Factor Analysis (CFA). Since one factor CBI was not confirmed, construct validity was examined using Exploratory Factor Analysis (EFA). The final number of factors was confirmed using CFA.

**Results:**

The internal consistency of the instrument was good with Cronbach’s alpha 0.89. Based on EFA, the CBI were loaded on two factors, eigenvalues >1, no item was removed. The emergent factors were named "Communicating respectfully" and "Professional knowledge and skill". These two factors explained 50.197% of the total variance. Then, CFA showed an acceptable fit for the two factors CBI.

**Conclusion:**

The results showed that the Persian version of the 16-item CBI had adequate validity and reliability. Accordingly, this instrument can be used to study nurses’ caring behaviors.

## Introduction

Nursing care is the foundation of the nursing profession that nurses promote professional behavior by communicating appropriately, providing support, demonstrating mutual respect, responsibility, and accountability [1]. Nursing care refers to a series of purposeful actions and attitudes of nurses to meet patients’ needs and reduce their pain and discomfort. Appropriate nursing behaviors allow nurses to demonstrate their professional competence to patients [2]. Patients’ perceptions of these behaviors significantly influence their satisfaction with the quality of care provided [3]. According to Papastavrou et al, patients’ perception of nursing behaviors influences their satisfaction more than therapeutic interventions [4].

Various factors such as patients’ social structure, lifestyle, culture [5] and interest, as well as their biographical, social, and physiological characteristics, may influence their perception of nurses’ caring behaviors [2], because caring behaviors are an interactive and mental process between patients and nurses [4]. As a result, patients as care recipients perceive caregivers to care behaviors differently, which may lead to care recipients’ feelings of unmet needs and dissatisfaction with the quality of care [3]. Given the importance of caregiver behaviors, an appropriate instrument is needed to examine patients’ perceptions of these behaviors [4].

Several instruments have been developed to examine patients’ perceptions of caregiving behaviors. One of them is the Caring Behaviors Inventory (CBI), which was proposed by Wolf et al. (1998) to measure nurses’ caring behaviors [1, 6, 7]. The CBI is a reflective instrument to assess nurses’ caring behaviors from the patients’ perspective. Behaviors is one of the most essential aspects in the evaluation of nursing care quality [4, 7].

The latest version of the CBI has 16 items represented by a factor. The score ranges from 16 to 96, and a higher score indicates better caring behavior [8]. This instrument has shown high validity and reliability in the United States [4, 9, 10] and has been used in several countries, including the United States, England [4, 10, 11], Japan, China, Panama, Italy, and Mexico [4, 12]. However, few studies have been conducted on its transcultural translation and psychometric analysis [4, 8–10, 12, 13].

In Iran, Rafiei et al. investigated the validity and reliability of the 42-item nurses’ CBI [14]. However, the transcultural translation of the CBI and psychometric analysis were not specifically studied. Atashzadeh et al. investigated the face validity of the 42-item nurses’ CBI in Iranian Population; they also reported good reliability for the inventory with Cronbach’s alpha of 0.81 [6]. But the 16 item CBI which measures the same concept with a smaller number of items and a lower burden on participants has not been validated in the Iranian population [8].

Wolf et al., at 2017, reduced the CBI to 16 items with one factor. The 16-item CBI includes four subscales: communicating respectfully (items 1, 2, 3, 4, 7, 8 and 10), ensuring human presence (item 11), communication, and positive attitude (items 5, 6, 9, 12, and 15), and professional knowledge and skills (items 13, 14, and 16), (Table 1). Convergent validity was assessed by analyzing the correlation between 24- and 16-item instruments, and a high correlation was found (n = 210, r = 0.99, p = 0.01). Excellent reliability was also reported with a Cronbach’s alpha coefficient of 0.95. Wolf et al. suggested that the instrument needs to be validated in different cultures [8]. However, the 16-item inventory has not been validated in Iranian population so far. Due to the reduction in the number of items and changes in the inventory, the objectives of this study are (1) to translate of the 16-item inventory transculturally and (2) to investigate the validity of this instrument for measuring nurses’ caring behaviors.

**Table 1:** Subscales of the 16-item CBI.

CBI items	CBI subscales
1. Carefully listens to you.	Communicating respectfully
2. Guides (helps) and teaches you.	
3. Take care of you by considering your individual preferences.	
4. Spending time with you.	
7. Being confident with you.	
8. Demonstrate skill and professional knowledge.	
10. Keep your information confidential.	
11. Voluntarily check up on you.	Ensuring human presence
5. Support you.	Communication & positive attitude
6. Demonstrate compassion with you.	
9. Include you in planning your care.	
12. Talk to you.	
15. Provide treatments and medications on time.	
13. Fulfill your spoken and unspoken needs.	Professional knowledge and skill
14. Respond rapidly to your request.	
16. Relieve your symptoms.	

## Methods

### Study design and population

This study was a cross-sectional study. Patients were enrolled in the medical departments of medical education centers affiliated with medical universities in different regions of Iran. Inclusion criteria were to have a history of at least one hospitalization, to be 18 years or older, and to be time and place oriented.

#### Measures

The demographic questionnaire collected data on the patient’s age, sex, education level, and place, time, and frequency of hospitalization. The second questionnaire, the CBI with 16 items, measured four subscales: communicating respectfully (items 1, 2, 3, 4, 7, 8 and 10), ensuring human presence (item 11), communication, positive attitude (items 5, 6, 9, 12, and 15), and professional knowledge and skill (items 13, 14, and 16). The items were answered on a 6-point Likert scale, and the total score ranged from 16 to 96 [8].

#### Sample size

To check validity, it is suggested to have 10 to 50 cases for each item [15, 16], so 30 cases per item were considered (480 cases), with 40 percent sampling loss, 700 cases were recruited. The online link of this inventory was sent by mobile phone number to the 700 patients who were admitted in different hospitals in Iran. Of the 700 patients 528 patients responded, and 509 patients completed the questionnaires in full.

### Data collection

In view of the cultural differences in Iran, the country has been divided into five distinctive regions: Center, North, South, West, and East. Three states were selected from each region, from each state, three cities, and from each city, ten hospitals were chosen randomly. Contact number of patients who met the inclusion criteria were obtained from each hospital. We spoke with the patients by telephone during their inpatient stay and invited them to complete an online questionnaire. CBI had not been used previously in electronic format before this study.

The online questionnaires were distributed via a link sent to the mobile phones of the 700 patients who agreed to participate in our study. The participants’ recruitment took place between May and November 2020. Participants may be representative of the low- and middle-income adult population in Iran.

### Transcultural translation and psychometrics analysis

This study was conducted in two parts: transcultural translation and psychometric analysis.

#### Transcultural translation

After obtaining permission from the original designer of the tool, the transcultural translation process was performed based on the Polit approach, which includes seven steps. The seven steps are translating the tool from English to Persian, combining original translations, translating the final version translated from Persian to English, reviewing the translated version from Persian to English, conducting a pilot study, modifying and summarizing, and testing the translated questionnaire [17]. Two experienced translators fluent in English with Persian mother language and expertise in healthcare services first translated the questionnaire into Persian, and then both versions were compared and combined. The translated tool was distributed among 10 patients to find potential ambiguities and problems. After the necessary corrections, the Persian translation of the inventory was re-translated to English by two bilingual people. Then, the last version was sent to the tool designer and got approved after a few edits.

#### Psychometric analysis

In the next step, face validity (qualitative), content validity (quantitative and qualitative), and construct validity were examined. In qualitative face validity, patients’ points of view on the level of difficulty, relevance, and ambiguity were examined, and the necessary corrections were made. To confirm the face validity, the inventory was administered to 10 patients, who were hospitalized in different wards of hospitals to express their ideas about simplicity and comprehensibility of each word and item. After incorporating a few comments given by the participants via interview, the items were edited.

To assess the content validity, ten experts (in the relevant instruments and caring behaviors) were asked to provide comments on grammar, use of appropriate words, proper placement of phrases, and appropriate scoring. Content Validity Index (CVI) and Content Validity Ratio (CVR) were then calculated to assess the quantitative content validity. Ten nursing experts were asked to rate each item to rate the CVR using the following equation [18]: CVR =) n_e_-N/2)/(N/2).

CVR score ranges from -1 to 1, and a score above zero indicates that the item is essential. The minimum acceptable score of CVR is checked using the Lawshe table, and items with a score lower than the minimum acceptable score are removed [19]. The CVI was calculated using the above equation. Items with CVI above 0.79 were retained.

Assumptions of the CFA and EFA were checked before doing the analysis. The fit of the samples was evaluated using the KMO index, which was equal to 0.92. The Bartlett test was then performed. Bartlett’s test was significant with Chi-square 3201.64 and P <0.001. Therefore, the samples had the necessary fitness and the minimum requirements to perform EFA.

Construct validity was then assessed by confirmatory factor analysis to confirm one factor CBI mentioned previously by the inventory’s designer. The one factor was not confirmed by our CFA. In the next step, we did an exploratory factor analysis to find the number of factors in the inventory. Maximum-Likelihood Estimation (MLE) method with varimax rotation was used to evaluate the analysis. The fit of the sample was confirmed using the Kaiser-Meyer-Olkin (KMO) index and Bartlett’s test.

In the next step, the final number of factors was confirmed using Confirmatory Factor Analysis (CFA). To judge the model fit, Comparative Fit Index (CFI), Standardized Root Mean Squared Residual (SRMSR), and Root Mean Squared Error of Approximation (RSMEA) were considered with cut points of > 0.95, < 0.06, and < 0.08, respectively [20].

The reliability of the tool was assessed before data collection. The reliability of the tool was assessed by the coefficient of intra-class coefficient correlation and Cronbach’s alpha. Cronbach’s alpha lower than 0.3 is considered low reliability, between 0.3–0.7 fair, and more than 0.7 is considered as good reliability [21, 22].

Analyses were conducted using IBM SPSS Statistics, Version 20 (IBM Corp., Armonk, NY) and LISREL (Linear Structural RELations) software version 8.80 (By Karl G. Joreskog & Dog Sorbom., Lincolnwood, IL 60712, USA). At last stage, to ensure the correct naming of the two factors, correspondence was checked with Dr. Wolf (who designed the original CBI), and approved by her.

### Ethical considerations

This study was approved by the ethic committee of Shahid Beheshti University of Medical Sciences (IR.SBMU.RETECH.REC.1399.108) and hospital directors were agreed to be part of the study. After calling patients and explaining the study, informed written consent was obtained from each. They participated in the study voluntarily and could leave the study at any stage.

## Results

The mean age of participants was 43.04±16.89. The demographic characteristics of the participants are presented in Table 2.

**Table 2:** Demographic characteristics of the participants.

	Range	Number	Percentage
**Age**	18	19	3.8
	18–27	83	16.5
	28–37	104	20.7
	38–47	89	17.7
	48–57	99	19.7
	58–67	71	14.1
	67–78	27	5.4
	<78	11	2.1
**Gender**	Male	251	49.9
	Female	252	50.1
**Education**	Primary education	70	13.9
	High school and lower	137	27.2
	High School Diploma	173	34.4
	college education	123	24.5
**Residency**	Urban	358	71.2
	Rural	145	28.8
**Number of hospitalization days**	1_5	394	78.3
	6_10	64	12.7
	11_15	19	3.8
	16–20	9	1.8
	21_25	4	0.8
	26_30	2	0.4
	31_35	5	1.0
	>35	6	1.2

In the qualitative phase, the face validity assessment items 3 and 7 were modified based on patients’ opinions. Item 3 was "Take care of you exceptionally," and item 7 was "Trustable to you" that item 3 was modified to "Take care of you by considering your individual preferences" and the item 7 was modified to "Being confident with you". These modified items then got approved by the designer. The mean CVI for difficulty, ambiguity, relevance were respectively, 0.88, 0.87, and 0.92. The overall mean CVR was 0.91. CVR and CVI for each item are reported in Table 3.

**Table 3:** The 16-item CBI and the reported CVI and CVR.

	CVI			CVR
Items	Difficulty	Ambiguity	Relevance	Necessary
1. Carefully listens to you	0.90	0.88	0.93	1.00
2. Guides (helps) and teaches you	0.88	0.87	0.93	1.00
3. Take care of you by considering your individual preferences.	0.79	0.80	0.95	0.71
4. Spending time with you.	0.95	0.88	0.88	1.00
5. Support you	0.88	0.90	0.93	0.86
6. Demonstrate compassion with you.	0.83	0.88	0.95	0.86
7. Being confident with you.	0.79	0.80	0.85	0.86
8. Demonstrate skill and professional knowledge	0.90	0.85	0.97	1.00
9. Include you in planning your care.	0.90	0.88	0.92	0.86
10. Keep your information confidential.	0.85	0.90	0.90	0.86
11. Voluntarily check up on you	0.88	0.93	0.92	0.86
12. Talk to you	0.90	0.87	0.88	1.00
13. Fulfill your spoken and unspoken needs	0.88	0.90	0.92	0.86
14. Respond rapidly to your request.	0.93	0.88	0.93	0.86
15. Provide treatments and medications on time.	0.85	0.87	0.92	1.00
16. Relieve your symptoms.	0.88	0.85	0.97	1.00
**Overall Mean**	0.88	0.87	0.92	0.91

**Fig 1:**
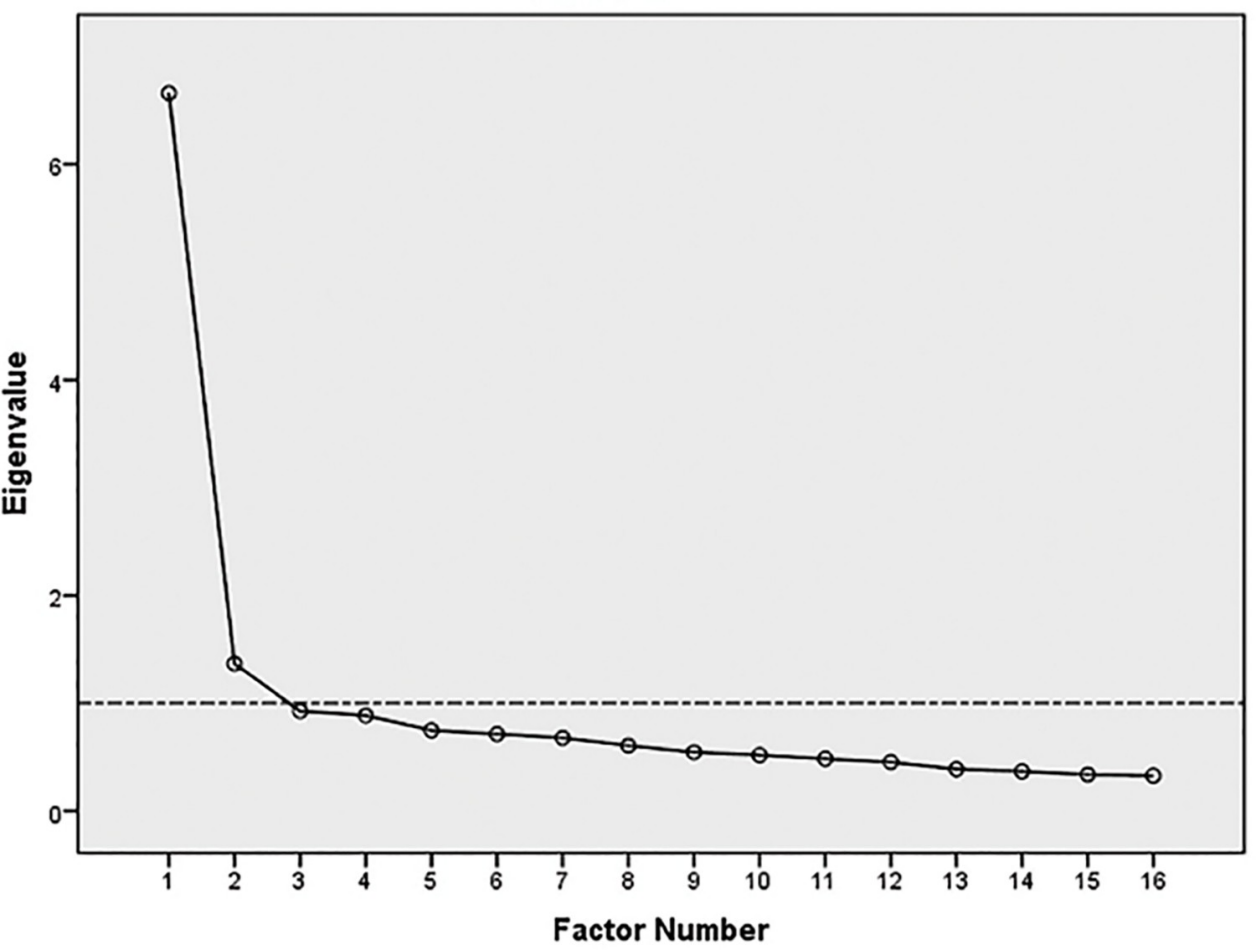
Scree plot of EFA for the 16-item CBI.

To check the construct validity of the tool with one factor CFA was done, but the one factor did not get confirmed by Chi-square /df = 595.71 / 104 = 5.73 with P = 0.0 and other indexes. CFI = 0.94, NFI = 0.93, GFI = 0.86, RMSEA = 0.10. Therefore, the construct validity was investigated using EFA. Based on Eigenvalues >1 and Scree Plot, two factors were extracted (Fig 1).

The two extracted factors were explaining 50.17% of the total variances, and no item was removed from the CBI. We named the two factors: "Communicating respectfully" and "Professional knowledge and skill". Items 3, 14, 15, and 16 were loaded on the factor "Communicating respectfully", and other items were loaded on the factor "Professional knowledge and skill" (Table 4).

**Table 4:** CBI exploratory factor analysis.

	Component	
	1	2
2. Guides (helps) and teaches you	0.75	0.14
3. Take care of you by considering your individual preferences.	0.71	0.18
4. Spending time with you	0.70	0.14
5. Support you	0.67	0.23
6. Demonstrate compassion with you.	0.67	0.25
1. Carefully listens to you	0.67	0.12
9. Include you in planning your care.	0.60	0.29
10. Keep your information confidential.	0.59	0.34
12. Talk to you	0.55	0.31
8. Demonstrate skill and professional knowledge	0.49	0.43
7. Being confident with you	0.49	0.44
15. Provide treatments and medications on time.	0.07	0.81
14. Respond rapidly to your request.	0.21	0.75
16. Relieve your symptoms.	0.20	0.74
13. Fulfill your spoken and unspoken needs	0.36	0.53
11. Voluntarily check up on you	0.48	0.50

Note: Extraction Method: Principal Component Analysis. Rotation Method: Varimax with Kaiser Normalization. Rotation converged in 3 iterations.

Then, the result of the EFA was tested using the CFA, and the fit indices showed acceptable model, CFI = 0.96, NFI = 0.95, GFI = 0.90, RMSEA = 0.08 (Table 5).

**Table 5:** Fit indices for one and two factor model CFA.

	χ^2^	df	χ^2^/df	P- value	CFI	NFI	GFI	RMSEA	SRMR
**Original Model (1 factors)**	595.71	104	5.73	<0.001	0.94	0.93	0.86	0.10	0.06
**Modified Model (2 factors)**	453	103	4.39	<0.001	0.96	0.95	0.90	0.08	0.05

Cronbach’s alpha was 0.89, indicating that the tool had good reliability. Cronbach’s alpha was also equal to α = 0.88 for the subscale "Communicating respectfully" and α = 0.78 for the subscale "Professional knowledge and skill" indicating good reliability and internal consistency for both subscales. The intra-class coefficient correlation for test-retest reliability for subscales and total scale were 0.94, 0.87, and 0.91, respectively.

The mean score of nurses’ caring behavior from patients’ point of view was 19.96 ± 2.79 for the "Communicating respectfully" factor and 59.05 ± 7.56 for the "Professional knowledge and skill" factor. Range of scores for the "Communicating respectfully" factors were 5–30, and 11–66 for the "Professional knowledge and skill" factor.

## Discussion

Validity and reliability are key indicators of any tool [17, 23]. In this study, the validity and reliability of CBI in the Persian language and Iranian culture were examined. The face validity indicated that the questions were simple and unambiguous.

According to EFA findings, 16 items were loaded on two factors based on eigenvalue, cumulative variance, and the scree plot assessments [22]. The "Communicating respectfully" (11 items) and "Professional knowledge and skill" (5 items) were the emergent factors. The construct validity of the tool was validated by the two factors CFA, and its reliability was confirmed by Cronbach’s alpha.

The psychometrics of the CBI with different items and emergent factors has been assessed in various countries. In Greece, for example, Papastavrou et al. provided a translation and psychometric analysis of the 24-item inventory with 245 patients. The results showed that the 24-item CBI had high validity with 4 factors: Assurance (8 items), Knowledge and skill (5 items), Respectful (6 items), and Connectedness (5 items). The reliability of the tool was reported good with Cronbach’s alpha of 0.93 for the overall 24-item CBI, 0.87 for Factor 1, Assurance, 0.76 for Factor 2, Knowledge and skill, 0.87 for Factor 3, Respectful, and 0.82 for Factor 4 –Connectedness [4]. Sarafis et al. (2016) also considered the validated 24-item nurses’ CBI to be suitable for use in Greece [24].

Wu et al. conducted a study to validate the CBI and to reduce the number of items. He reduced the 42 items to a 24-item CBI. The 24-item CBI was validated, and its reliability in measuring nurses’ caring behaviors was reported as good and cost-effective [10]. In reducing the 24-item tool to the 16-item, Wolf et al. concluded that the 16-item CBI with one factor explained up to 58% of the total variance. Sub-scales in the 16-item with one factor included communicating respectfully (items 1, 2, 3, 4, 7, 8, and 10), ensuring human presence (item 11), communication, and positive attitude (items 5, 6, 9, 12, and 15), and professional knowledge and skill (items 13, 14, and 16). Convergent validity was assessed using correlation analysis between 24- and 16-item tools (n = 210, r = 0.99, p <0.01) [10], which was high, and the authors considered the use of this tool cost-effective [10], which is consistent with the present study. The emergent factors "Communicating respectfully" and "Professional knowledge and skill" in this study are in line with other studies and emphasize the need for respectful communication with the patient and having the necessary skills and knowledge.

Gul and Dinc performed psychometric analysis on the 42-item CBI in Turkey and concluded that a 30-item with three factors CBI had high validity and good reliability. The emergent factors in their study included the "Respectful Deference to Others, "Professional Knowledge and Attitude for nurses," and Professional Knowledge and Attitude for patients". They reported Cronbach’s alpha of 0.99 for the 30-item CBI with three factors. Also, they reported Cronbach’s alpha of 0.98 for "Respectful Deference to Others", 0.99 for "Professional Knowledge and Attitude for nurses", and 0.98 for Professional Knowledge and Attitude for patients". They recommended a psychometric analysis of the above tools in other cultures [13].

The two factors of communicating respectfully and professional knowledge and skill were identified in all the above studies [8, 13, 24], and their importance was emphasized. In this study, both factors are emphasized. In this study, knowledge, and skills were one of the dimensions of the tool that was obtained in similar studies. Therefore, it can be concluded that nurses’ knowledge and skills are the most important components affecting care delivery. Therefore, it is recommended that training programs be planned and implemented according to the needs of nurses working in each ward. Since the other dimension in the present study is communication skills, it is necessary to pay more attention to improve nurses’ ability to communicate properly.

Given the different number of factors in different countries, it can be concluded that culture affects patients’ perceptions of nurses’ caring behavior. The results of this study indicated that the 16-item inventory has high validity and reliability in Iran and can be used as a suitable tool to investigate the nurses’ caring behavior. Also, it has a lower participant burden than the 42-item CBI due to the small number of items. However, due to the impact of environment and culture on patients’ perceptions of nurses’ caring behavior, the Persian version of the 16-item tool is proposed to study nurses’ caring behavior in Iran, and its translation and psychometric analysis should be done for other cultures.

One of the strengths of this research was the sufficient number of participants from the different educational and medical hospitals in Iran. The limitation of this study was the completion of questionnaires online, which did not allow for careful examination of participants and monitoring the completion of questionnaires. Another limitation of this study was the incidence of COVID 19 disease, which affects the number of study variables. Discussion about findings of this study is difficult for researchers because there is not any study about transcultural and validation of CBI-16 in any language.

## Conclusion

The CBI with two factors and 16 items has high reliability and can be used as a suitable tool to study the nurses’ caring behavior in Iran.

## Supporting information

S1 File Persian version of CBI. (DOCX)

S2 File CBI manuscript data. (SAV)

## List of abbreviations

CBI: Caring Behaviors Inventory
CVR: content validity ratio
CVI: content validity index
KMO: Kaiser-Meyer-Olkin
MLE: Maximum-Likelihood Estimation
EFA: Exploratory Factor Analysis
CFA: Confirmatory Factor Analysis
RMSEA: Root Mean Square Error of Approximation
CFI: Comparative Fit Index
NFI: Normed Fit Index
GFI: Goodness of Fit Index
AGFI: Adjusted Goodness of Fit Index
