# Supplementary material for: Translation and validation of caring behaviors inventory among nurses in Iran
Source: PLoS One. 2021 Jul 9;16(7):e0254317. doi: 10.1371/journal.pone.0254317 (PMC8270114; doi:10.1371/journal.pone.0254317)
Supplement: S1 File — (DOCX) [file pone.0254317.s001.docx]

| **هرگز** | **تقریباً هرگز** | **گاهی اوقات** | **معمولاً** | **تقریبا‌ً همیشه** | **همیشه** | **عبارت فارسی** |
| --- | --- | --- | --- | --- | --- | --- |
|  |  |  |  |  |  | 1. به حرف‌های شما با دقت گوش می‌دهند. |
|  |  |  |  |  |  | 2. به شما آموزش می‌دهند و شما را راهنمایی مي‌كنند |
|  |  |  |  |  |  | 3. از شما به خوبی مراقبت می‌کنند (یعنی در مراقبت از شما خواسته‌هایتان را در نظر می‌گیرند). |
|  |  |  |  |  |  | 4. با صبر و حوصله از شما مراقبت می­کنند. |
|  |  |  |  |  |  | 5. شما را حمایت می‌کنند. |
|  |  |  |  |  |  | 6. با شما مهربان/ شفیق هستند. |
|  |  |  |  |  |  | 7. به آن‌ها اطمینان داريد. |
|  |  |  |  |  |  | 8. در كار خود دانش و مهارت دارند. |
|  |  |  |  |  |  | 9. از شما می‌خواهند در برنامه مراقبتی‌تان همکاری و مشارکت کنید. |
|  |  |  |  |  |  | 10. رازدار هستند و اطلاعات شما را فاش نمی‌کنند. |
|  |  |  |  |  |  | 11. با شوق و خوشرويي با شما رفتار مي‌كنند. |
|  |  |  |  |  |  | 12. با شما حرف زده و گفتگو می‌کنند. |
|  |  |  |  |  |  | 13. در صورت درخواست و ياعدم درخواست نیازهاي شما را برآورده می‌کنند. |
|  |  |  |  |  |  | 14. در هنگام زنگ زدن/ صدا کردن به سرعت به شما پاسخ می‌دهند. |
|  |  |  |  |  |  | 15. داروهای شما را سر وقت می‌دهند و کارهای شما را به‌موقع انجام می‌دهند. |
|  |  |  |  |  |  | 16. درد و ناراحتی شما را تسکین می‌دهند. |

سیاهه رفتارهای مراقبتی

لطفا عبارات زیر که بیانگر رفتارهای مراقبتی پرستاران است، را بخوانید و در ستون توصیف کننده رفتار پرستار/ پرستاران علامت ضربدر بگذارید.
